# A new optimization algorithm to solve multi-objective problems

**DOI:** 10.1038/s41598-021-99617-x

**Published:** 2021-10-13

**Authors:** Mohammad Reza Sharifi, Saeid Akbarifard, Kourosh Qaderi, Mohamad Reza Madadi

**Affiliations:** 1 Department of Hydrology and Water Resources, Faculty of Water and Environmental Engineering, Shahid Chamran University of Ahvaz, Ahvaz, Iran; 2 Department of Water Engineering, Faculty of Agriculture, Shahid Bahonar University of Kerman, Kerman, Iran; 3 Department of Water Engineering, Faculty of Agriculture, University of Jiroft, Jiroft, Iran

**Keywords:** Engineering, Mathematics and computing

## Abstract

Simultaneous optimization of several competing objectives requires increasing the capability of optimization algorithms. This paper proposes the multi-objective moth swarm algorithm, for the first time, to solve various multi-objective problems. In the proposed algorithm, a new definition for pathfinder moths and moonlight was proposed to enhance the synchronization capability as well as to maintain a good spread of non-dominated solutions. In addition, the crowding-distance mechanism was employed to select the most efficient solutions within the population. This mechanism indicates the distribution of non-dominated solutions around a particular non-dominated solution. Accordingly, a set of non-dominated solutions obtained by the proposed multi-objective algorithm is kept in an archive to be used later for improving its exploratory capability. The capability of the proposed MOMSA was investigated by a set of multi-objective benchmark problems having 7 to 30 dimensions. The results were compared with three well-known meta-heuristics of multi-objective evolutionary algorithm based on decomposition (MOEA/D), Pareto envelope-based selection algorithm *II* (PESA-II), and multi-objective ant lion optimizer (MOALO). Four metrics of generational distance (*GD*), spacing (*S*), spread (Δ), and maximum spread (*MS*) were employed for comparison purposes. The qualitative and quantitative results indicated the superior performance and the higher capability of the proposed MOMSA algorithm over the other algorithms. The MOMSA algorithm with the average values of CPU time = 2771 s, *GD* = 0.138, *S* = 0.063, Δ = 1.053, and *MS* = 0.878 proved to be a robust and reliable model for multi-objective optimization.

## Introduction

Today, most of the engineering problems require dealing with multiple conflicting objectives instead of a single-objective. For such problems, the multi-objective optimization (MOO) is an efficient technique for finding a set of solutions that define the best tradeoff between competing objectives while satisfying several criteria. MOO was introduced by Vilfredo Pareto and today, it became an important tool for decision-making in many fields of engineering, where the optimal decisions should be taken between conflict objectives. Various methods have been proposed for the MOO. In 1984, Schaffer introduced the innovative idea of employing evolutionary optimization algorithms for multi-objective optimization^1^. Since then, several researchers have attempted to develop different types of multi-objective evolutionary algorithms^2–12^. The several advantages of the evolutionary optimization algorithms such as the gradient-free mechanism and the local optima avoidance have made them readily applicable to real problems in different fields of science.

The literature shows that the multi-objective evolutionary algorithms are able to efficiently approximate the true Pareto optimal solutions of multi-objective problems. However, in 1997, Wolpert and Macready, by proposing the No Free Lunch-NFL theorem, claimed that there is no optimization technique capable to solve all optimization problems^13^. According to this theorem, the superior performance of an optimization method in a category of problems cannot guarantee its’ superiority on another category of problems. This theorem encourages the researchers to propose new optimization evolutionary algorithms for new categories of problems in the study field.

Some of the most well-known multi-objective evolutionary algorithms which have had desirable performance in solving large scale engineering problems are Strength–Pareto Evolutionary Algorithm, SPEA^14,15^, Non-dominated Sorting Genetic Algorithm, NSGA^16^, Non-dominated Sorting Genetic Algorithm version 2, NSGA-II^2^, Multi-Objective Particle Swarm Optimization, MOPSO^17^, Multi-Objective Evolutionary Algorithm based on Decomposition, MOEA/D^18^, Pareto Archived Evolution Strategy, PAES^19^, and Pareto–frontier Differential Evolution, PDE^20^, Multi-Objective Water Cycle Algorithm, MOWCA^21^, and Multi-Objective Grey Wolf Optimizer, MOGWO^22^, Pareto envelope-based selection algorithm-II, PESA-II^23^ and Multi-Objective Ant Lion Optimizer, MOALO^24^.

All the aforementioned multi-objective algorithms are indeed the developed version of their single-objective algorithms. One of the strongest single-objective algorithms within the family of evolutionary algorithms is the moth swarm algorithm (MSA). The MSA has been proven to be superior to over 80 other evolutionary algorithms^25–33^. Due to the novelty of the MSA, there is still no study in the literature to design a multi-objective version of this algorithm. Accordingly, this paper proposes the multi-objective moth swarm algorithm (MOMSA), for the first time, in order to optimize the problems with multiple objectives. The proposed algorithm was tested on 7 multi-objective benchmark problems having 7 to 30 dimensions and the results were compared with three well-known meta-heuristics of MOEA/D, PESA-II and MOALO.

The rest of the paper is organized as follows. “Methodology” section presents a brief overview on the single-objective MSA, and next the modeling procedure of the multi-objective MSA followed by the utilized performance metrics. Next, the utilized benchmark functions (ZDT and DTLZ) are introduced and thereafter the comparative multi-objective algorithms are briefly introduced. The last part of “Methodology” section is devoted to the sensitivity analysis on the algorithms parameters. “Results and discussion” section provides the qualitative and qualitative results as well as relevant discussion. Eventually, “Conclusion” section concludes the work and outlines some recommendations for researchers.

## Methodology

### Single-objective MSA

Single-objective MSA, proposed by Mohamed et al.^25^, was inspired by the behavior of moths in the nature. Here, a brief overview on this algorithm is provided. More details and the mathematical explanations can be found in Mohamed et al.^25^.

In the MSA, three groups of moths (pathfinders, prospectors, and onlookers) and a light source are considered. Pathfinders are capable to find the best position over the optimization space with First-In, Last-Out principle to guide the movement of the main swarm. Prospectors tend to wander into a random spiral path nearby the light sources, which have been marked by the pathfinders. Onlookers drift directly toward the best global solution (moonlight), which has been achieved by prospectors’ moths. Therefore, the possible solution in MSA is represented by position of light source, and the quality of this solution is considered as luminescence intensity of the light source.

The MSA algorithm is performed through three phases of initialization, reconnaissance, and transverse orientation. At the beginning of the flight, the position of each moths (initial solution) is randomly determined by a randomization function (initialization phase). Then, the type of each moth in the population is selected based on the fitness value (objective function). The best moth is considered as pathfinder (light sources) and the best and worst groups of moths are considered as prospectors and onlookers, respectively. During the prospecting process, the moths may be concentrated in some parts of the response space, led to entrapment in the local optima and reducing the quality of some moth populations. To prevent premature convergence and improve the diversity in solutions, a part of the moth population is required to prospect the areas with less swarm. Pathfinders are responsible for this role. Thus, they update their position through interaction with each other and crossover operations and with the ability to fly long distances (known as lévy mutation) and prevent the stop in local optima (reconnaissance phase). The flight path of moths toward a light source can be described by cone-shaped logarithmic spirals. Accordingly, a set of paths located on the surface of the cone, with a fixed central angle, can describe the flight path of moths to the light source. A group of the moth with the highest luminescence intensities is selected as the prospectors. The number of prospectors should be reduced in each iteration (transverse orientation phase).

During the optimization process in the MSA, by reducing the number of prospectors, the number of onlookers increases. This leads to a faster convergence to the global solution. The increased convergence speed is in fact, due to the celestial navigation. An onlooker moth with the lowest luminescence can travel directly toward the best solution (moon). Hence, to control the recent movement, this step of the MSA algorithm is designed such a way that onlookers are forced to search more effectively through focusing on important points of prospector. To this purpose, the onlookers are divided into two parts with Gaussian walk and associative learning mechanism. In the MSA, the type of each moth is alternately varied. Thus, each prospector that provides a better solution (greater luminescence than the light source) is promoted to the pathfinder. At the end of each step, the new light and moonlight sources will be available as possible solutions.

The problem-solving steps of the single-objective MSA algorithm are shown by Algorithm 1 .
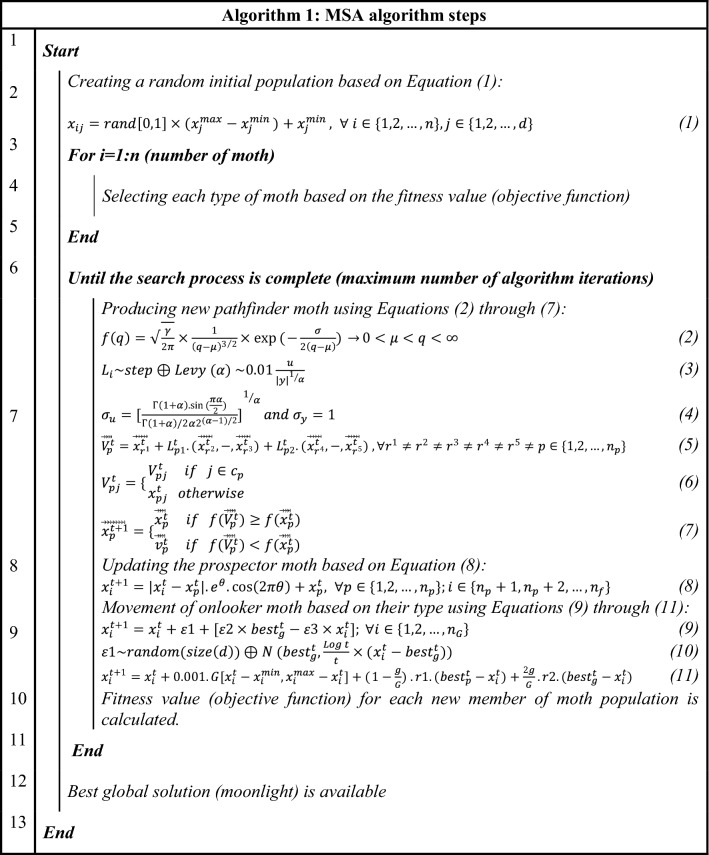


### Mathematical model of multi-objective problems

Optimizing multi-objective problems (MOPs) involves more than one objective function that should be optimized simultaneously. Equation (12) expresses the mathematical form of the objective function of the MOP problem.

12 $$F\left(X\right)={[{F}_{1}\left(X\right), {F}_{2}\left(X\right), \dots , {F}_{m}\left(X\right)]}^{T},$$

where $X={x}_{1}, {x}_{2}, {x}_{3}, \dots , {x}_{d}$ is the vector of variables, and d and m refer to the number of variables and objectives, respectively.

The most common solution for MOPs is to keep a set of the best solutions in an archive and update it per iteration. In this method, the best solutions are defined as non-dominant solutions or Pareto optimal solutions. A solution can be considered as a non-dominant solution if and only if the following conditions are met^34^:

- Pareto dominance: $U=\left(u1, u2, u3,\dots , un\right)<V=(v1, v2, v3,\dots ,vn)$ if and only if *U* is slightly lower than *V* in the objective space, which means:
  13 $$\left\{\begin{array}{c}{f}_{i}\left(U\right)\le {f}_{i}\left(V\right) \forall i\\ {f}_{i}\left(U\right)<{f}_{i}\left(V\right) \exists i\end{array}\right. \quad i=1, 2, 3,\dots , m.$$
- Pareto optimal solution: *U* vector is an optimal solution if and only if none of the other solutions can dominate *U*. A set of Pareto optimal solutions is called Pareto optimal front (*PF*_*optimal*_).

**Figure 1:**
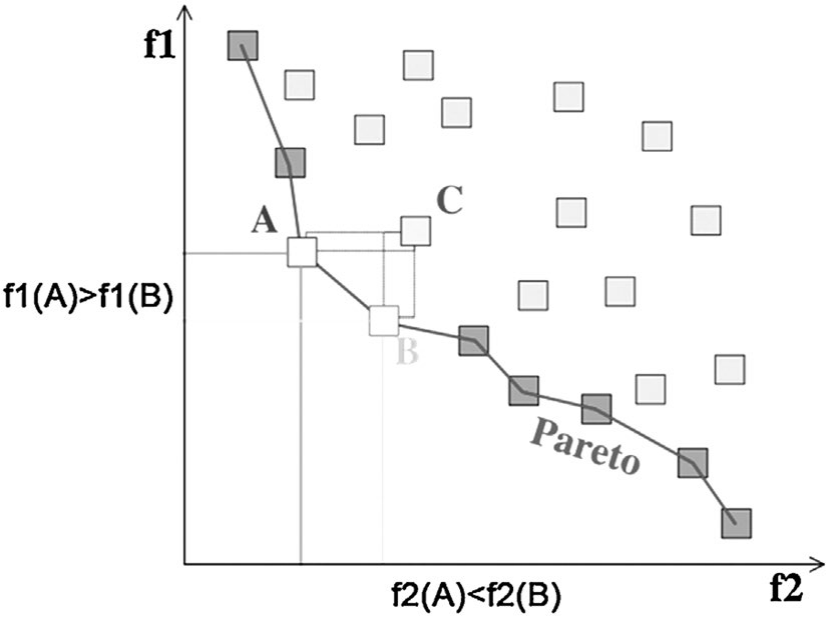
Pareto optimal solutions (A and B) for 2D space.

Figure 1 shows that of the three solutions A, B, and C, the solution C has the maximum values for *f*1 and *f*2; as a result, it is considered the dominant solution. On the contrary, both solutions A and B can be taken into account as non-dominant solutions.

### Multi-objective MSA

**Figure 2:**
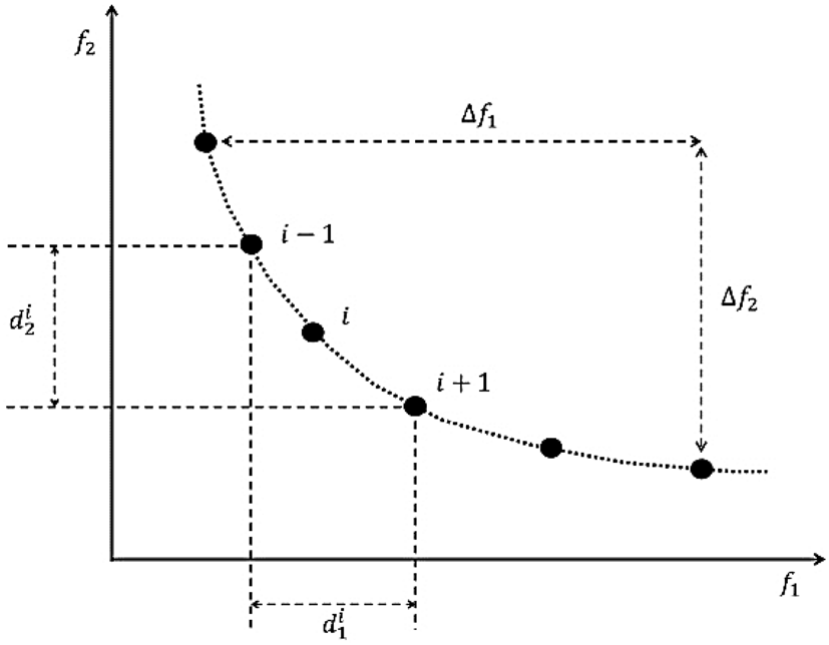
Calculating crowding-distance for point *i* (moth).

In order to develop the single-objective MSA to an efficient multi-objective optimization algorithm, the dominant features of the algorithm must be properly defined. Accordingly, the definition for selecting the type of moths and the best value (moonlight) should be changed to a multi-objective space. In this way, the crowding-distance mechanism was employed to select the most efficient (best) solutions in the population as the pathfinder moths and moonlight (global optimum). This mechanism shows the distribution of non-dominant solutions around a non-dominant solution. Figure 2 shows the calculation of crowding-distance for point *i* (moth) from the Pareto front.

The crowding-distance for a moth can also be calculated by the following equations:

14 $${\Delta }_{i}=\sum_{j=1}^{{N}_{O}}\frac{{d}_{j}^{i}}{{\Delta f}_{j}},$$

15 $${\Delta f}_{j}=\left|{f}_{j}^{max}-{f}_{j}^{min}\right| , j\in \left[1, {N}_{O}\right],$$

16 $${d}_{j}^{i}=\left|{f}_{j}^{i+1}-{f}_{j}^{i-1}\right| , i\in {\widehat{X}}_{f}^{l},$$

where ${N}_{O}$ refers to the number of problem objective functions, ${\widehat{X}}_{f}^{l}$ refers to the Pareto front, ${f}_{j}^{max}$ and ${f}_{j}^{min}$ are the maximum and minimum values obtained for the jth objective function, and ${f}_{j}^{i+1}$ and ${f}_{j}^{i-1}$ are the values for the *j*th objective function for *i* + 1 and *i − *1 moths.

A lower crowding-distance value shows a greater distribution of solutions in a specific region. In multi-objective problems (MOPs), this parameter is calculated in the objective spaces; hence, all the non-dominant solutions must be classified based on the values for one of the objective functions. These parameters should be calculated for each of the non-dominant solutions. An important step in the MOMSA is the selection of the moonlight and pathfinders from the population, as the best solution in each iteration. This affects the MOMSA synchronization capability and retains a good spread of non-dominant solutions.

The crowding-distance must be calculated for all non-dominant solutions and in all iterations of the algorithm. It is essential to determine the solutions having the highest crowding-distance values. Then, the non-dominant solutions are considered as the moonlight and the pathfinders. In addition, the prospectors and onlookers are determined based on the crowding-distance values. Some non-dominant solutions are made in proximity to the moonlight and pathfinders in the next iteration, and their distance values are reduced and declined.

It is essential to reserve the non-dominant solutions in an archive to achieve the Pareto front sets. This archive gets updated with each iteration and the dominated solutions are eliminated from the archive. Therefore, whenever the number of members in the Pareto archive becomes larger than the size of Pareto archive, the crowding-distance is used to eliminate the non-dominant solutions that have the lowest crowding-distance values among the Pareto archive members. MOMSA has a great potential for the exploitation in the design space, it focuses on the near-optimal solutions and exploits the long-distance solutions.

Furthermore, the Lévy mutation and the collaborative learning mechanisms were used to improve the exploitation and exploration capabilities of the developed algorithm, respectively. MOMSA usually starts with the exploitation phase, the prospectors move toward the pathfinders and the onlookers move toward the prospectors. However, in the early iteration, these motions act as a heuristic factor. This trend can be considered as the MOMSA capability to find a wide range of design space, while focusing on the optimal non-dominant solutions.

In MOMSA, a simple approach is defined to handle the limitations of MOPs. After obtaining a set of solutions in each iteration, all the limitations are investigated and some solutions in the possible space are selected. Then, the non-dominant solutions are selected from the possible solutions and are imported into the Pareto archive. Finally, the moonlight and pathfinders are selected from this archive for the next iteration. Different problem-solving steps of the MOMSA algorithm are shown in Algorithm 2. 
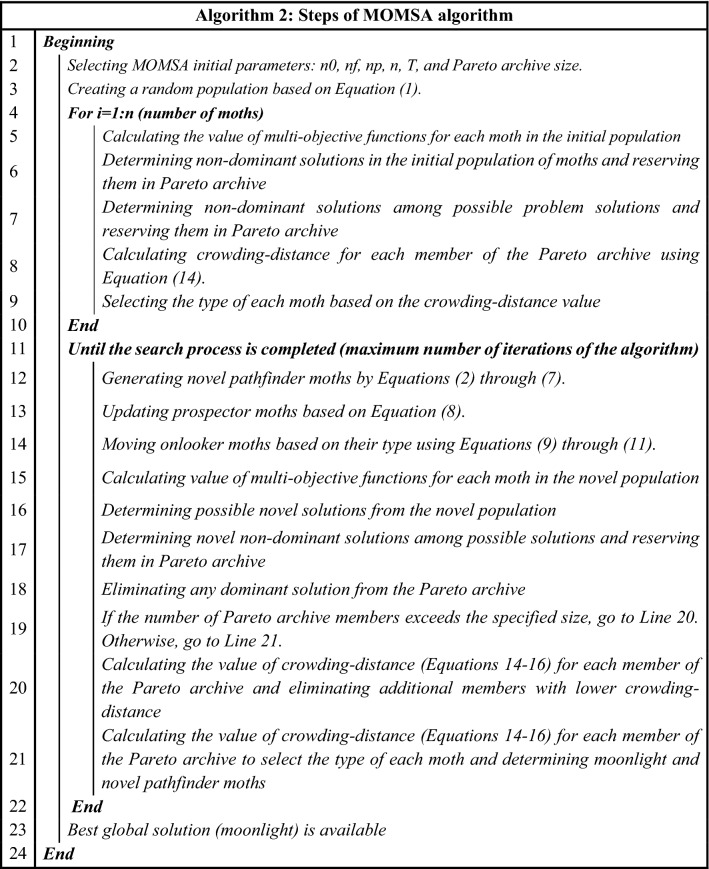


### Performance metrics

In order to evaluate the performance of multi-objective evolutionary algorithms in optimization of multi-objective problems, four performance metrics of generational distance (*GD*), metric of spacing (*S*), metric of spread (Δ), and maximum spread (*MS*) were used. Here, these metrics are briefly introduced.

**Figure 3:**
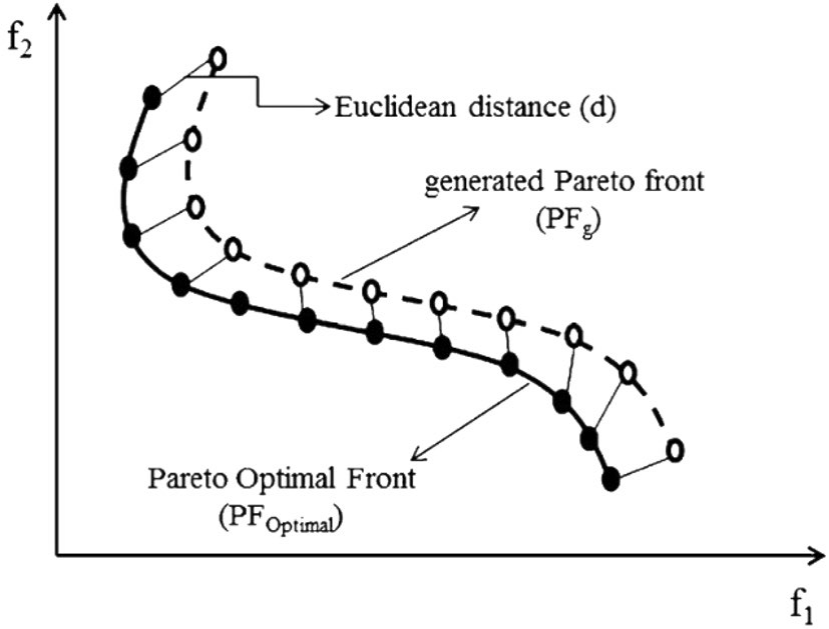
Schematic of the GD metric for MOPs^35^.

*GD,* defined by Van Veldhuizen and Lamont^35^, refers to the distance between the generated Pareto front with the Pareto optimal front. This metric determines the ability of an algorithms to find a set of non-dominant solutions that has the lowest distance to the Pareto optimal front. In other words, the algorithms with the lowest *GD* have the best convergence with the Pareto optimal front. *GD* is calculated by Eq. (17)^36^:

17 $$GD={\frac{1}{NPF}\left(\sum_{i=1}^{NPF}{d}_{i}^{2}\right)}^\frac{1}{2},$$

where *NPF* is the number of members in the generated Pareto front (*PF*) and *d* is the Euclidean distance between *ith* member in *PF*_*g*_ and the nearest member in *PF*_*optimal*_. Figure 3 shows a schematic view of the *GD* metric in 2D space. The best metric derived for *GD* is zero, so that *PF*_*g*_ is exactly on *PF*_*optimal*_.

**Figure 4:**
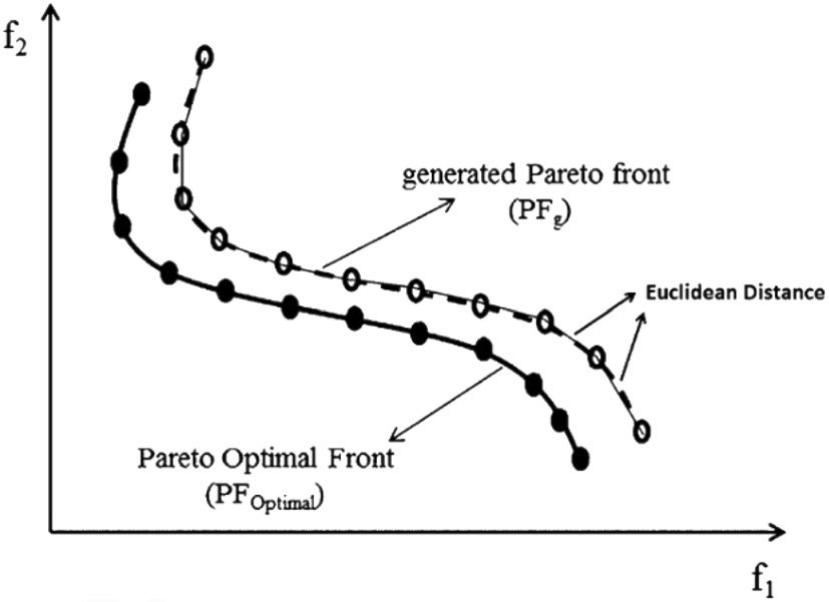
A schematic view of *S* metric for MOPs^37^.

*S* refers to the metric of spacing which indicates the distribution of non-dominated solutions obtained by an algorithm. This metric shows how well the obtained solutions are distributed among each other^37^. This metric is calculated by Eq. (18)^38^:

18 $$S=\sqrt{{\frac{1}{NPF-1}\sum_{i=1}^{NPF}({d}_{i}-\overline{d })}^{2},}$$

where ${d}_{i}={min}_{j}(\left|{f}_{1}^{i}\left(x\right)-{f}_{1}^{j}\left(x\right)\right|+\left|{f}_{2}^{i}\left(x\right)-{f}_{2}^{j}\left(x\right)\right|$ , *i,j* = 1,2,…,NPF, and $\overline{d }$ is the mean of all *d*_*i*_. The lowest value of *S* leads to the best uniform distribution in *PF*_*g*_. If all the non-dominant solutions are evenly distributed in *PF*_*g*_, the values of *d*_*i*_ and *d* are the same; as a result, the value of *S* is equal to zero. Figure 4 shows a schematic view of *S* metric.

**Figure 5:**
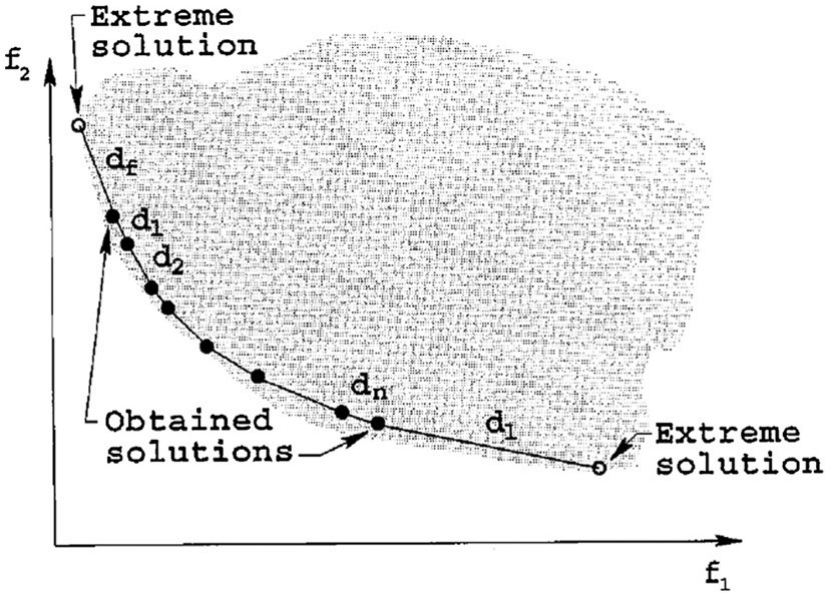
Schematic view of metric of spread (Δ) for MOPs^2,38^.

Metric of spread (Δ) shows the extent of spread attained by the non-dominated solutions obtained from an algorithm^38^. It is calculated by Eq. (19)^38^:

19 $$\Delta =\frac{{d}_{f}+{d}_{l}+\sum_{i=1}^{NPF}\left|{d}_{i}-\overline{d }\right|}{{d}_{f}+{d}_{l}+(NPF-1)\overline{d} },$$

where *d*_*f*_ and *d*_*l*_ are the Euclidean distance between the extreme solutions (starting and ending points) in *PF*_*optimal*_ and *PF*_*g*_ respectively, and *d*_*i*_ refers to the distance between each point in *PF*_*g*_ and the closest point in *PF*_*optimal*_. The value of Δ is always larger than zero and its lower value means the best distribution and extension of solutions. If Δ is equal to zero, the excellent conditions occur, indicating that extreme solutions of *PF*_*optimal*_ have been found and that ${d}_{i}=\overline{d }$ for all non-dominated points. Figure 5 shows a schematic view of Δ metric for a Pareto optimal front.

Maximum spread (MS) shows how much the starting and ending points of *PF* line overlap the similar points on the *PF*_*optimal*_ line; hence, the proximity of the two extreme points in *PF*_*optimal*_ and *PF*_*g*_ is measured. In other words, this metric shows how much the lines of discovered non-dominant solutions overlap the Pareto line; therefore, the greater overlapping, the better. This metric is defined as Eq. (20)^39^:

20 $$MS={\left[\frac{1}{m}\sum_{i=1}^{m}{\left[\frac{\mathrm{min}\left({f}_{i}^{max},{F}_{i}^{max}\right)-\mathrm{max}({f}_{i}^{min},{F}_{i}^{min})}{{F}_{i}^{max}-{F}_{i}^{min}}\right]}^{2}\right]}^\frac{1}{2},$$

where ${f}_{i}^{max}$ and ${f}_{i}^{min}$ are the maximum and minimum values of the *i*th objective in *PF*_*g*_, respectively, and ${F}_{i}^{max}$ and ${F}_{i}^{min}$ are the maximum and minimum values of the *i*th objective in *PF*_*optimal*_, respectively. A larger value of *MS* refers to better expansion of solutions.

### Benchmark problems

In order to evaluate the capability and performance of the developed MOMSA algorithm, which was coded in the programming section of MATLAB R2014a software (www.mathworks.com), several standard multi-objective benchmark functions were used. These problems were different types of multi-objective problems (MOPs) with various features that were selected from a set of valid studies. In all the benchmark functions, in order to achieve reliable results, 10 independent runs of each algorithm were compared. In all these cases, the mean of the best results was shown. Moreover, for appropriate and fair comparison with the other algorithms, the number of iteration and the size of the Pareto archive for all MOPs were considered as the same and determined by the sensitivity analysis.

#### ZDT benchmark problems

The ZDT test suite, created by Zitzler et al.^39^ consists of six test problems. It is the most widely employed suite of benchmark multi-objective problems in the EA literature. Table 1 shows the ZDT standard bi-objective benchmark functions with different dimensions.

**Table 1:** ZDT multi-objective test function suite^39^ (Incidentally, due to being binary encoded, ZDT5 has often been omitted from analysis elsewhere in the EA literature.

Function name	d	Problem	Variable bounds	Pareto front
ZDT1	30	$OF1\left(x\right)={x}_{1}$ $OF2(x)=g(x)\left(1-\sqrt{\frac{OF1(x)}{g(x)}}\right)$ (21) $g(x)=1+\frac{9\sum_{i=2}^{d}{x}_{i}}{d-1}$	${x}_{i}\in \left[\mathrm{0,1}\right]$ $i=2,\dots ,d$	Convex
ZDT2	30	$OF1(x)={x}_{1}$ $OF2(x)=g(x){\left(1-\frac{OF1(x)}{g(x)}\right)}^{2}$ (22) $g(x)=1+\frac{9\sum_{i=2}^{d}{x}_{i}}{d-1}$	${x}_{i}\in \left[\mathrm{0,1}\right]$ $i=2,\dots ,d$	Non-convex
ZDT3	30	$OF1(x)={x}_{1}$ $OF2(x)=g\left[1-\sqrt{\frac{OF1\left(x\right)}{g\left(x\right)}}-\frac{OF1\left(x\right)}{g\left(x\right)}\mathrm{sin}(10\pi {x}_{i})\right]$ (23) $g(x)=1+\frac{9\sum_{i=2}^{d}{x}_{i}}{d-1}$	${x}_{i}\in \left[\mathrm{0,1}\right]$ $i=2,\dots ,d$	Convex, disconnected
ZDT4	10	$OF1(x)={x}_{1}$ $OF2(x)=g\left(1-\sqrt{\frac{OF1(x)}{g(x)}}\right)$ (24) $g(x)=1+10\left(d-1\right)+\sum_{i=2}^{d}\left[{x}_{i}^{2}-10\mathrm{cos}(4\pi {x}_{i})\right]$	${x}_{1}\in \left[\mathrm{0,1}\right]$ ${x}_{2},\dots ,{x}_{d}\in \left[-\mathrm{5,5}\right]$ $i=2,\dots ,d$	Non-convex
ZDT6	10	$OF1\left(x\right)=1-\mathrm{exp}(-4{x}_{1}){sin}^{6}(6\pi {x}_{1})$ $OF2\left(x\right)=g\left(x\right)\left[1-{\left(\frac{OF1(x)}{g(x)}\right)}^{2}\right]$ (25) $g(x)=1+9{\left(\frac{\sum_{i=2}^{d}{x}_{i}}{d-1}\right)}^{0.25}$	${x}_{i}\in \left[\mathrm{0,1}\right]$ $i=2,\dots ,d$	Non-convex, Nonuniformly spaced

#### DTLZ benchmark problems

The DTLZ test suite, created by Deb et al.^40^ is unlike the majority of multi-objective test problems in which the problems are scalable to any number of objectives. Table 2 shows DTLZ1 and DTLZ2 standard tri-objective benchmark functions with 7 and 12 dimensions.

**Table 2:** DTLZ1 and DTLZ2 tri-objective test function^39^.

Function name	d	Problem	Parameter domains	Pareto front
DTLZ1	7	${OF}_{1}=\left(1+g\right)0.5\prod_{i=1}^{M-1}{y}_{i}$ ${OF}_{m=2:M-1}=\left(1+g\right)0.5\left(\prod_{i=1}^{M-m}{y}_{i}\right)(1-{y}_{M-m+1})$ (26) ${OF}_{M}=\left(1+g\right)0.5(1-{y}_{1})$ $g=100\left[k+\sum_{i=1}^{k}({\left({z}_{i}-0.5\right)}^{2}-\mathrm{cos}\left(20\pi \left({z}_{i}-0.5\right)\right))\right]$	$\left[\mathrm{0,1}\right]$	Triangular-linear
DTLZ2	12	${OF}_{1}=\left(1+g\right)\prod_{i=1}^{M-1}\mathrm{cos}({y}_{i}\pi /2)$ ${OF}_{m=2:M-1}=\left(1+g\right)\left(\prod_{i=1}^{M-m}\mathrm{cos}({y}_{i}\pi /2)\right)sin({y}_{M-m+1}\pi /2)$(27) ${OF}_{M}=\left(1+g\right)sin({y}_{1}\pi /2)$ $g=\sum_{i=1}^{k}{\left({z}_{i}-0.5\right)}^{2}$	$\left[\mathrm{0,1}\right]$	Inverted-concave

### Comparative algorithms

To investigate the efficiency of the proposed MOMSA, the results were compared with three well-known multi-objective algorithms of MOEA/D, SPEA-II and MOALO.

The MOEA/D algorithm, proposed by Zhang and Li^18^, needs to decompose the target MOP. Zhang and Li^18^ used the Tchebycheff decomposition approach to serve this purpose. In MOEA/D, the population is composed of the best solution found so far for each subproblem. Only the current solutions to its neighboring subproblems are exploited for optimizing a subproblem in MOEA/D. The PESA-II algorithm, proposed by Corne et al.^23^, utilizes a selection technique for MOO algorithms in which the unit of selection is a hyper box in the objective space. In this technique, instead of assigning a selective fitness to an individual, the selective fitness is assigned to the hyper boxes in objective space which are currently occupied by at least one individual in the current approximation to the Pareto frontier. A hyper box is thereby selected, and the resulting selected individual is randomly chosen from this hyper box. This method of selection is shown to be more sensitive to ensuring a good spread of development along the Pareto frontier than individual-based selection. The MOALO algorithm, proposed by Mirjalili et al.^24^, was inspired by the mimics the hunting mechanism of antlions and the interaction of their favorite prey, ants, with them in nature. In this algorithm, a repository is first employed to store non-dominated Pareto optimal solutions obtained so far. Solutions are then chosen from this repository using a roulette wheel mechanism based on the coverage of solutions as antlions to guide ants towards promising regions of multi-objective search spaces.

### Sensitivity analysis

**Figure 6:**
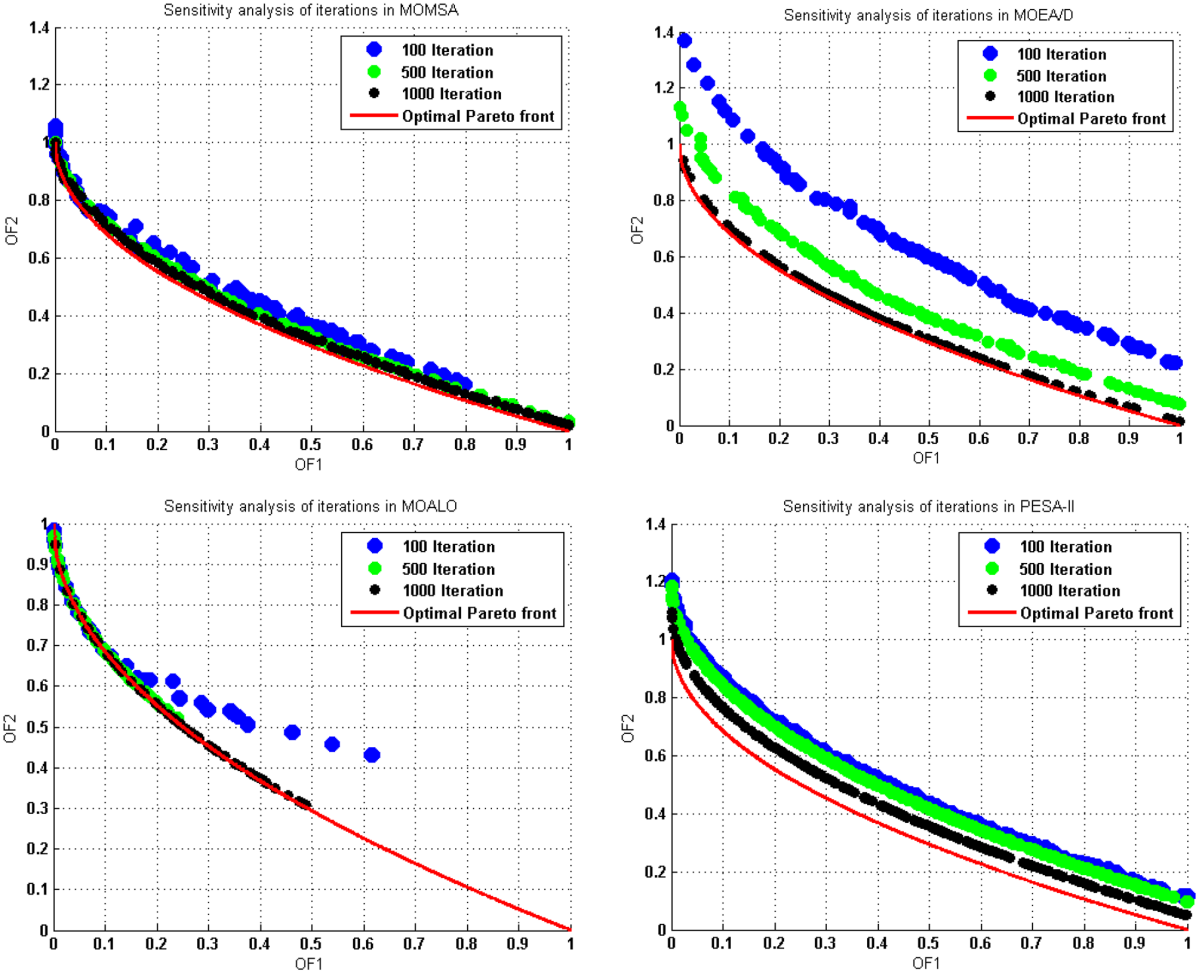
Sensitivity analysis on the number of iterations in the ZDT1 multi-objective test function.

Sensitivity analysis is an essential ingredient of MOO algorithms building and quality assurance. In this study, a sensitivity analysis was carried out to obtain the best values of the algorithm’s parameters. Here the results of the sensitivity analysis on the number of iterations for all the utilized algorithms in solving the ZDT1 benchmark function are presented. As seen in Fig. 6, by increasing the number of iterations, the obtained Pareto front gets closer to the optimal Pareto front, so that, after 1000 iterations, the obtained Pareto front has been largely overlapped to the optimal Pareto front.

The quantitative values of performance metrics at the Table 3 confirmed the above results. This table shows that after 1000 iterations, all the performance metrics of the utilized algorithms have reached their best values. Accordingly, the number of iterations was considered to be 1000 for all the algorithms.

**Table 3:** Statistical results for Sensitivity analysis on the number of iterations in the ZDT1 multi-objective test function.

Algorithm	MOMSA			MOEA/D		
Number of iterations	100 Iteration	500 Iteration	1000 Iteration	100 Iteration	500 Iteration	1000 Iteration
GD	0.062	0.026	0.017	0.244	0.075	0.008
S	0.062	0.056	0.044	0.059	0.054	0.049
Δ	0.972	0.754	0.581	1.514	1.008	0.865
MS	0.848	0.988	0.990	0.569	0.727	0.966

Algorithm	MOALO			PESA-II		
Number of iterations	100 Iteration	500 Iteration	1000 Iteration	100 Iteration	500 Iteration	1000 Iteration
GD	0.098	0.083	0.022	0.164	0.091	0.048
S	0.084	0.072	0.066	0.083	0.074	0.063
Δ	1.793	1.360	1.247	0.923	0.812	0.719
MS	0.272	0.341	0.586	0.807	0.851	0.972

Table 4 provides the best values of MOMSA, MOEA/D, MOALO, and PESA-II algorithms’ parameters based on the sensitivity analysis. In all the benchmark functions, in order to achieve reliable results, 10 independent runs of each algorithm were compared. Furthermore, to have a fair comparison, the size of Pareto archive was considered the same.

**Table 4:** Parameter setting of the MOMSA, MOEA/D, MOALO, and PESA-II algorithms in the multi-objective test functions.

Algorithms	Number of iterations	Number of design variables	Pareto front archive size
MOMSA	1000	7–10–12–30	100
MOEA/D	1000	7–10–12–30	100
MOALO	1000	7–10–12–30	100
PESA-II	1000	7–10–12–30	100

## Results and discussion

As mentioned in the introduction, this study proposes the multi-objective moth swarm algorithm to solve various multi-objective problems. In order to evaluate the performance of the developed MOMSA algorithm, four evaluation metrics of GD, S, Δ, and MS were used. The results of MOMSA were compared with three well-known algorithms of MOEA/D, PESA-II and MOALO. Tables 5, 6, 7 and 8 indicate the values of the performance metrics (for 10 independent runs) of the utilized multi-objective algorithms in optimization of ZDT bi-objective and DTLZ tri-objective benchmark functions.

**Table 5:** Statistical results for GD evaluation metrics on ZDT bi-objective and DTLZ tri-objective test function suite.

Function		ZDT1 (bi-objective)				ZDT2 (bi-objective)				ZDT3 (bi-objective)				ZDT4 (bi-objective)			
Index	Algorithm	MOMSA	MOEA/D	MOALO	PESA-II	MOMSA	MOEA/D	MOALO	PESA-II	MOMSA	MOEA/D	MOALO	PESA-II	MOMSA	MOEA/D	MOALO	PESA-II
GD	Worst	0.048	0.224	0.080	0.109	0.056	0.098	0.052	0.109	0.024	0.431	0.056	0.171	0.668	9.818	42.430	28.987
	Best	0.017	0.008	0.022	0.048	0.007	0.003	0.017	0.075	0.012	0.087	0.008	0.051	0.137	6.176	6.031	7.140
	Average	**0.028**	0.159	0.044	0.079	**0.029**	0.033	0.033	0.095	**0.017**	0.220	0.028	0.087	**0.391**	7.901	21.070	17.466
	Std. dev.	0.014	0.102	0.026	0.026	0.024	0.043	0.015	0.015	0.005	0.150	0.022	0.056	0.294	1.504	16.942	10.931
CPU time		2386 s	2512 s	3375 s	2693 s	2961 s	3079 s	3755 s	3090 s	2821 s	2842 s	3240 s	2909 s	1274 s	1371 s	1624 s	1595 s

Function		ZDT6 (bi-objective)				DTLZ1 (tri-objective)				DTLZ2 (tri-objective)							
Index	Algorithm	MOMSA	MOEA/D	MOALO	PESA-II	MOMSA	MOEA/D	MOALO	PESA-II	MOMSA	MOEA/D	MOALO	PESA-II				
GD	Worst	0.083	0.441	0.537	1.117	1.490	11.945	34.165	30.017	0.067	0.070	0.050	0.163				
	Best	0.030	0.018	0.058	0.052	0.013	3.468	26.593	22.179	0.009	0.007	0.019	0.128				
	Average	**0.063**	0.131	0.325	0.560	**0.411**	6.490	30.775	26.767	0.029	**0.023**	0.031	0.148				
	Std. dev.	0.024	0.207	0.198	0.464	0.720	3.869	3.570	3.296	0.026	0.031	0.015	0.016				
CPU time		1579 s	1726s	1953s	2393 s	4194 s	4312 s	4846 s	4574 s	4184 s	4215 s	4581 s	4438 s				

The bold values in the table represnt the best values obtained by the utilized algorithms for each benchmark function.

**Table 6:** Statistical results for S evaluation metrics on ZDT bi-objective and DTLZ tri-objective test function suite.

Function		ZDT1 (bi-objective)				ZDT2 (bi-objective)				ZDT3 (bi-objective)				ZDT4 (bi-objective)			
Index	Algorithm	MOMSA	MOEA/D	MOALO	PESA-II	MOMSA	MOEA/D	MOALO	PESA-II	MOMSA	MOEA/D	MOALO	PESA-II	MOMSA	MOEA/D	MOALO	PESA-II
S	Worst	0.054	0.055	0.076	0.075	0.077	0.062	0.064	0.062	0.156	0.263	0.213	0.255	0.101	0.688	0.278	0.558
	Best	0.044	0.049	0.066	0.063	0.040	0.056	0.024	0.052	0.147	0.129	0.124	0.234	0.047	0.166	0.081	0.160
	Average	**0.050**	0.051	0.071	0.068	0.055	0.059	**0.044**	0.056	**0.151**	0.169	0.171	0.244	**0.076**	0.336	0.146	0.365
	Std. dev.	0.004	0.003	0.004	0.006	0.017	0.003	0.022	0.004	0.004	0.063	0.041	0.010	0.024	0.238	0.091	0.195

Function		ZDT6 (bi-objective)				DTLZ1 (tri-objective)				DTLZ2 (tri-objective)							
Index	Algorithm	MOMSA	MOEA/D	MOALO	PESA-II	MOMSA	MOEA/D	MOALO	PESA-II	MOMSA	MOEA/D	MOALO	PESA-II				
S	Worst	0.077	0.059	0.095	0.555	0.051	0.404	1.394	14.942	0.016	0.215	0.081	0.117				
	Best	0.045	0.036	0.063	0.072	0.014	0.008	0.552	2.723	0.007	0.186	0.012	0.087				
	Average	0.059	**0.045**	0.080	0.268	**0.036**	0.120	0.954	8.475	**0.011**	0.204	0.062	0.100				
	Std. dev.	0.016	0.009	0.014	0.204	0.018	0.190	0.415	5.016	0.004	0.012	0.033	0.014				

The bold values in the table represnt the best values obtained by the utilized algorithms for each benchmark function.

**Table 7:** Statistical results for Δ evaluation metrics on ZDT bi-objective and DTLZ tri-objective test function suite.

Function		ZDT1 (bi-objective)				ZDT2 (bi-objective)				ZDT3 (bi-objective)				ZDT4 (bi-objective)			
Index	Algorithm	MOMSA	MOEA/D	MOALO	PESA-II	MOMSA	MOEA/D	MOALO	PESA-II	MOMSA	MOEA/D	MOALO	PESA-II	MOMSA	MOEA/D	MOALO	PESA-II
Δ	Worst	0.910	1.180	1.443	0.831	1.167	1.086	1.198	0.806	0.941	1.383	1.658	1.007	1.298	1.191	1.018	0.996
	Best	0.581	0.865	1.247	0.719	0.858	0.978	1.006	0.647	0.872	1.288	1.476	0.916	0.921	1.132	1.001	0.936
	Average	**0.696**	1.044	1.345	0.763	1.011	1.045	1.126	**0.721**	**0.910**	1.330	1.552	0.958	1.102	1.161	1.008	**0.973**
	Std. dev.	0.148	0.133	0.081	0.052	0.153	0.048	0.091	0.065	0.033	0.044	0.082	0.037	0.160	0.031	0.008	0.029

Function		ZDT6 (bi-objective)				DTLZ1 (tri-objective)				DTLZ2 (tri-objective)							
Index	Algorithm	MOMSA	MOEA/D	MOALO	PESA-II	MOMSA	MOEA/D	MOALO	PESA-II	MOMSA	MOEA/D	MOALO	PESA-II				
Δ	Worst	1.622	1.202	1.493	1.420	1.306	1.104	1.696	1.708	1.296	1.069	1.514	1.462				
	Best	1.209	1.081	1.382	0.969	0.933	0.973	1.412	1.066	0.954	0.991	1.455	0.631				
	Average	1.476	**1.137**	1.425	1.211	1.052	**1.042**	1.581	1.389	1.122	**1.019**	1.488	1.049				
	Std. dev.	0.195	0.052	0.053	0.190	0.172	0.062	0.120	0.262	0.163	0.034	0.024	0.348				

The bold values in the table represnt the best values obtained by the utilized algorithms for each benchmark function.

**Table 8:** Statistical results for MS evaluation metrics on ZDT bi-objective and DTLZ tri-objective test function suite.

Function		ZDT1 (bi-objective)				ZDT2 (bi-objective)				ZDT3 (bi-objective)				ZDT4 (bi-objective)			
Index	Algorithm	MOMSA	MOEA/D	MOALO	PESA-II	MOMSA	MOEA/D	MOALO	PESA-II	MOMSA	MOEA/D	MOALO	PESA-II	MOMSA	MOEA/D	MOALO	PESA-II
MS	Worst	0.978	0.819	0.359	0.834	0.944	0.926	0.130	0.947	0.922	0.718	0.625	0.784	0.719	0.317	0.197	0.328
	Best	0.990	0.966	0.586	0.972	0.947	0.998	0.327	0.962	0.966	0.962	0.960	0.981	0.850	0.815	0.472	0.696
	Average	**0.986**	0.924	0.499	0.916	0.945	**0.961**	0.254	0.953	**0.946**	0.847	0.835	0.881	**0.769**	0.571	0.309	0.531
	Std. dev.	0.006	0.070	0.104	0.059	0.001	0.033	0.089	0.006	0.019	0.127	0.151	0.080	0.056	0.227	0.133	0.152

Function		ZDT6 (bi-objective)				DTLZ1 (tri-objective)				DTLZ2 (tri-objective)							
Index	Algorithm	MOMSA	MOEA/D	MOALO	PESA-II	MOMSA	MOEA/D	MOALO	PESA-II	MOMSA	MOEA/D	MOALO	PESA-II				
MS	Worst	0.868	0.829	0.603	0.637	0.503	0.319	0.174	0.146	0.807	0.932	0.456	0.456				
	Best	0.878	0.946	0.899	0.726	0.928	0.668	0.400	0.256	0.987	0.999	0.626	0.626				
	Average	0.871	**0.887**	0.794	0.677	**0.724**	0.454	0.286	0.213	0.902	**0.964**	0.524	0.524				
	Std. dev.	0.005	0.060	0.133	0.036	0.209	0.153	0.112	0.053	0.095	0.032	0.072	0.072				

The bold values in the table represnt the best values obtained by the utilized algorithms for each benchmark function.

As can be seen, the developed MOMSA algorithm was superior in the majority of the standard bi-objective and tri-objective benchmark functions. In terms of CPU time, the proposed MOMSA algorithm had the lowest executing time for all the bi-objective and tri-objective benchmark functions. For example, for the ZDT6 bi-objective function, the CPU time for the MOMSA was 1579 s, while for the MOEAD, MOALO, and PESA-II, the CPU time was 1726s, 1953s, and 2393 s, respectively. Similar results were obtained for tri-objective functions. For example, for the DTLZ2 benchmark function, the CPU time for the MOMSA, MOEA/D, MOALO and PESA-II was 4184 s, 4215 s, 4581, and 4438 s, respectively. This indicates that the MOMSA was the fastest model in running the code, it can achieve impressive results in a much shorter time.

In terms of the *GD* metric, the MOMSA with the lowest *GD* outperformed in all the ZDT bi-objective benchmark functions. For example, the average value of *GD* for ZDT3 benchmark function obtained by the MOMSA algorithm was 0.017, while the corresponding values for MOEA/D, MOALO and PESA-II algorithms were 0.22, 0.028, and 0.087, respectively, indicating the higher performance of the MOMSA compared to the other algorithms. Therefore, it can be said that the MOMSA could find the non-dominant solutions with minimum distance from *PF*_*optimal*_ (GD metric) and had a better distribution than the three other algorithms. Also, for the tri-objective benchmark functions, the MOMSA though had the best results for DTLZ1, but for DTLZ2, it placed at the second rank after the MOEA/D algorithm.

For the *S* metric, while the MOMSA algorithm outperformed the other algorithms in optimizing the ZDT1, ZDT3 and ZDT6 benchmark functions, the MOALO and the MOEA/D algorithms demonstrated better results for the ZDT2 and ZDT6, respectively. In addition, for both the tri-objective standard functions (DTLZ1 and DTLZ2), the MOMSA was the superior model in terms of *S*.

The obtained results for Δ metric demonstrated that the MOMSA was superior for ZDT1 (Δ = 0.696) and ZDT3 (Δ = 0.910) functions, the MOEA/D was superior for ZDT6 (Δ = 1.137), DTLZ1 (Δ = 1.042) and DTLZ2 (Δ = 1.019) functions and the PESA-II produced the best results for ZDT2 (Δ = 0.721) and ZDT4 (Δ = 0.973) functions.

As seen in Table 8, the MOMSA outperforms the other algorithms for most of the benchmark functions in terms of *MS* metric, and the MOEA/D obtained the second rank. Overall, it is found from Tables 5, 6, 7 and 8 that the proposed MOMSA algorithm was superior to the three other studied algorithms, especially in *GD* and *S* metrics.

**Figure 7:**
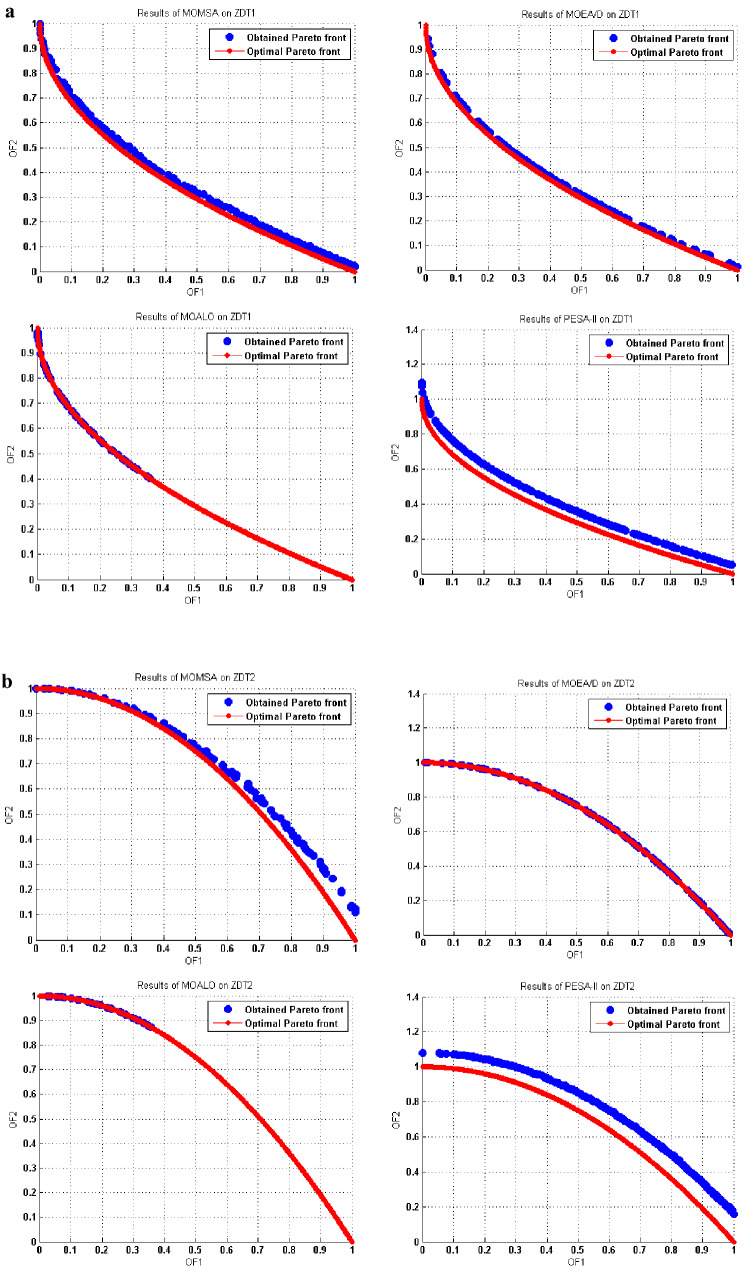

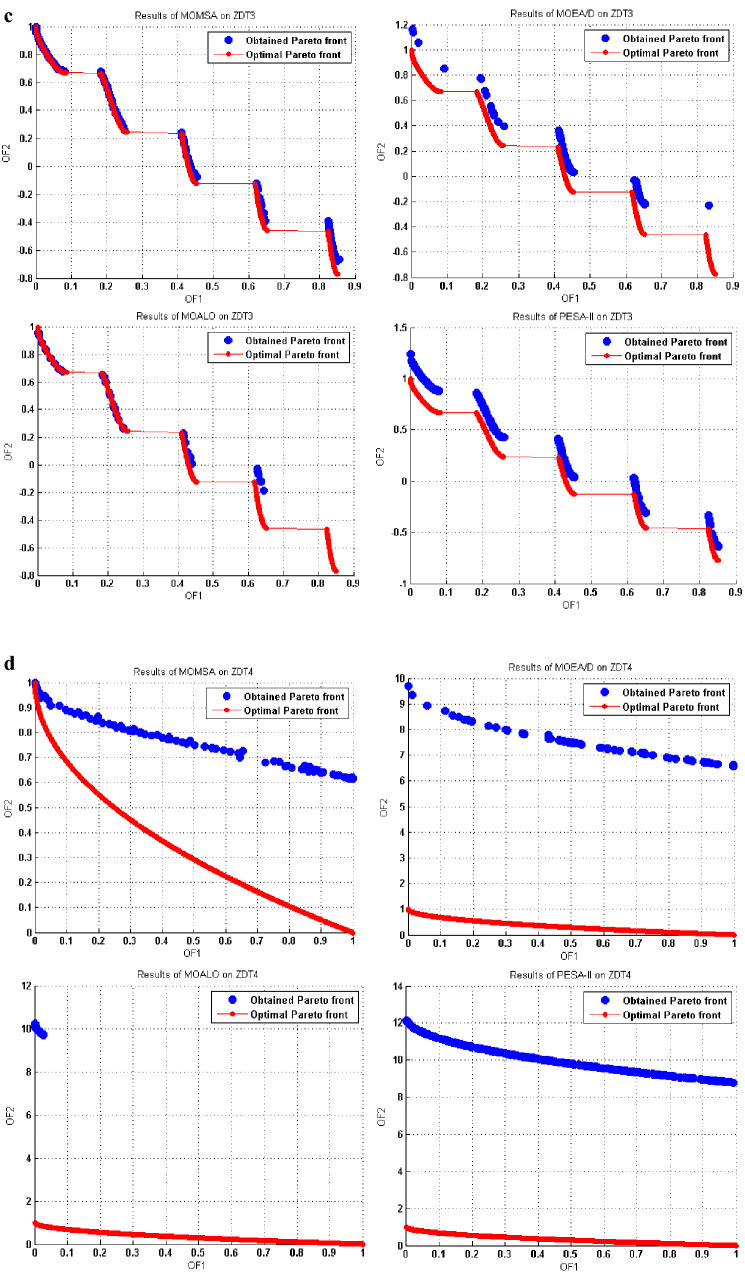

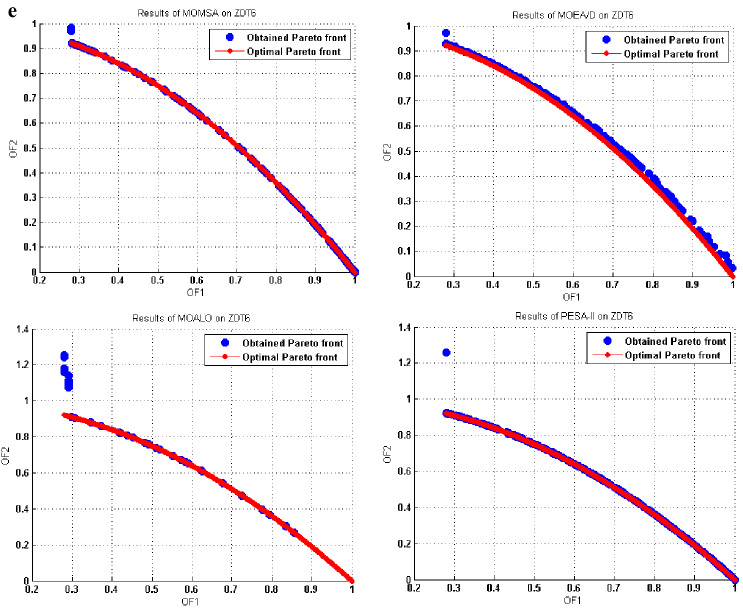
(**a**) Obtained Pareto optimal solutions by MOMSA, MOEA/D, MOALO, and PESA-II for ZDT1. (**b**) Obtained Pareto optimal solutions by MOMSA, MOEA/D, MOALO, and PESA-II for ZDT2. (**c**) Obtained Pareto optimal solutions by MOMSA, MOEA/D, MOALO, and PESA-II for ZDT3. (**d**) Obtained Pareto optimal solutions by MOMSA, MOEA/D, MOALO, and PESA-II for ZDT4. (**e**) Obtained Pareto optimal solutions by MOMSA, MOEA/D, MOALO, and PESA-II for ZDT6.

**Figure 8:**
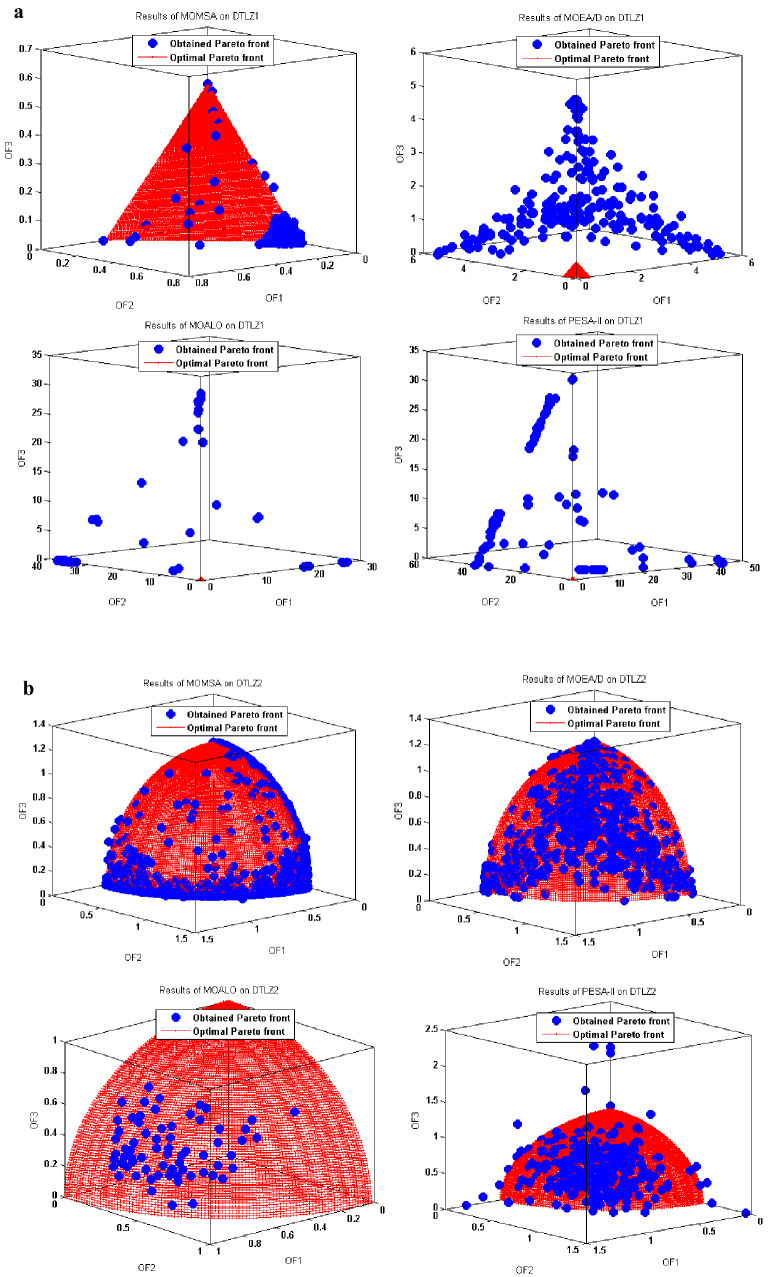
(**a**) Obtained Pareto optimal solutions by MOMSA, MOEA/D, MOALO, and PESA-II for DTLZ1. (**b**) Obtained Pareto optimal solutions by MOMSA, MOEA/D, MOALO, and PESA-II for DTLZ2.

This superiority was also more evident in Figs. 7 and 8. These figures show a graphical comparison between the true (optimal) and the obtained Pareto fronts by the multi-objective algorithms in solving the ZDT and DTLZ standard multi-objective benchmark functions.

For ZDT1 and ZDT2 benchmark function, two algorithms of MOMSA and MOEA/D yielded more accurate results, very close to the optimal Pareto front, but the MOALO could not cover the optimal Pareto front and the SPEA-II failed to produce satisfactory distribution and spread for the non-dominated solutions. For ZDT3 benchmark function, the proposed MOMSA was the only model. In addition, the proposed MOMSA was much more successful than the MOEA/D in optimizing the ZDT4 benchmark function, while the SPEA-II and MOALO algorithms were unable to produce reasonable results. For ZDT6 benchmark function, the distributions of the obtained fronts are similar for all the algorithms to some extent, slightly better for the MOMSA algorithm.

Similar results were obtained for the tri-objective benchmark functions of DTLZ1 and DTLZ2. As seen in Fig. 8, the proposed MOMSA was the only model that had impressive results. It could produce a better distribution and spread for the non-dominated solutions compared to the MOEA/D. In this case, the performance of MOALO and the SPEA-II was not acceptable. For the DTLZ2 benchmark function, the Pareto front obtained by MOEA/D algorithm was closer to the optimal Pareto front compared to the proposed MOMSA. Nevertheless, the MOMSA also gave quite good results. Both the MOEA/D and MOMSA algorithms were much more successful than the MOALO and SPEA-II algorithms. Comparing the graph of SPEA-II with that of MOALO indicates the superiority of the former algorithm in optimization of DTLZ2 benchmark function. All these results were quantitatively obtained in the previous section.

**Figure 9:**
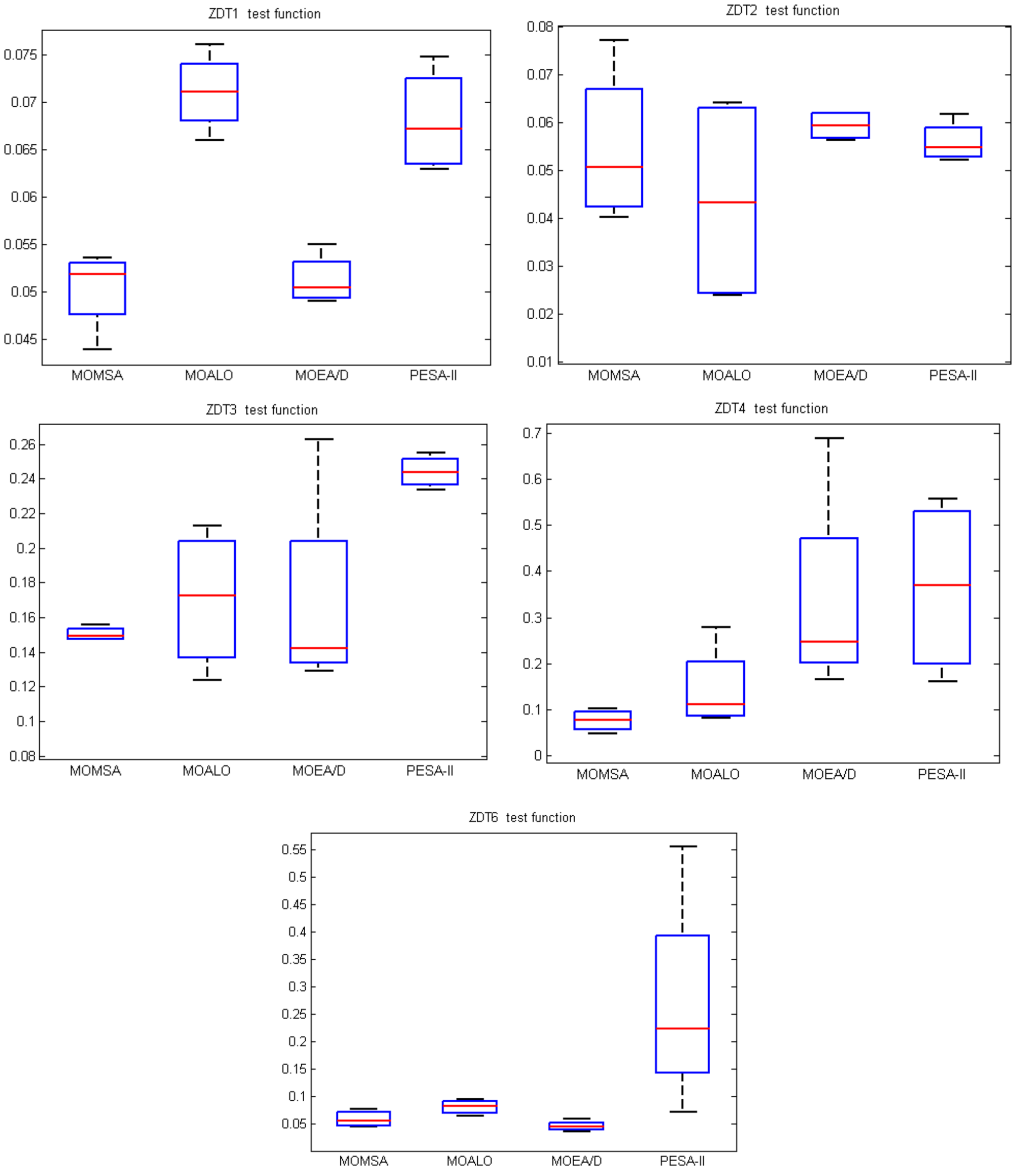
Boxplot of the statistical results for *S* evaluation metrics on ZDT bi-objective test function suite.

Figure 9 shows the boxplots of the metrics of spacing (S) derived from 10 independent runs of the MOMSA, MOEA/D, MOALO and SPEA-II algorithms in solving the ZDT multi-objective standard benchmark functions. As seen, the boxplots of the proposed MOMSA and the MOEA/D are narrower than the MOALO and SPEA-II for ZDT1, ZDT3 and ZDT6, indicating the superior performance of MOMSA and MOEA/D in solving those benchmark functions. These results prove that these two algorithms were able to provide remarkable convergence and coverage ability in solving multi-objective problems. For the ZDT2, the MOEA/D followed by PESA-II has narrower boxplots indicating the better efficiency of those algorithms compared to the MOMSA and MOALO. In ZDT4 benchmark problem, the MOMSA was the best model, it has the narrowest boxplot and located under the minima of MOALO, MOEA/D, and PESA-II algorithms. For ZDT6 benchmark function, the boxplot of all the algorithms are similar to some extent, slightly better for the MOEA/D algorithm. After that, the proposed MOMSA, and the MOALO placed in the next ranks. The results of PESA-II algorithm in solving the ZDT6 were poor. Similar results were observed for the three other performance metrics (*GD*, *MS* and Δ).

**Figure 10:**
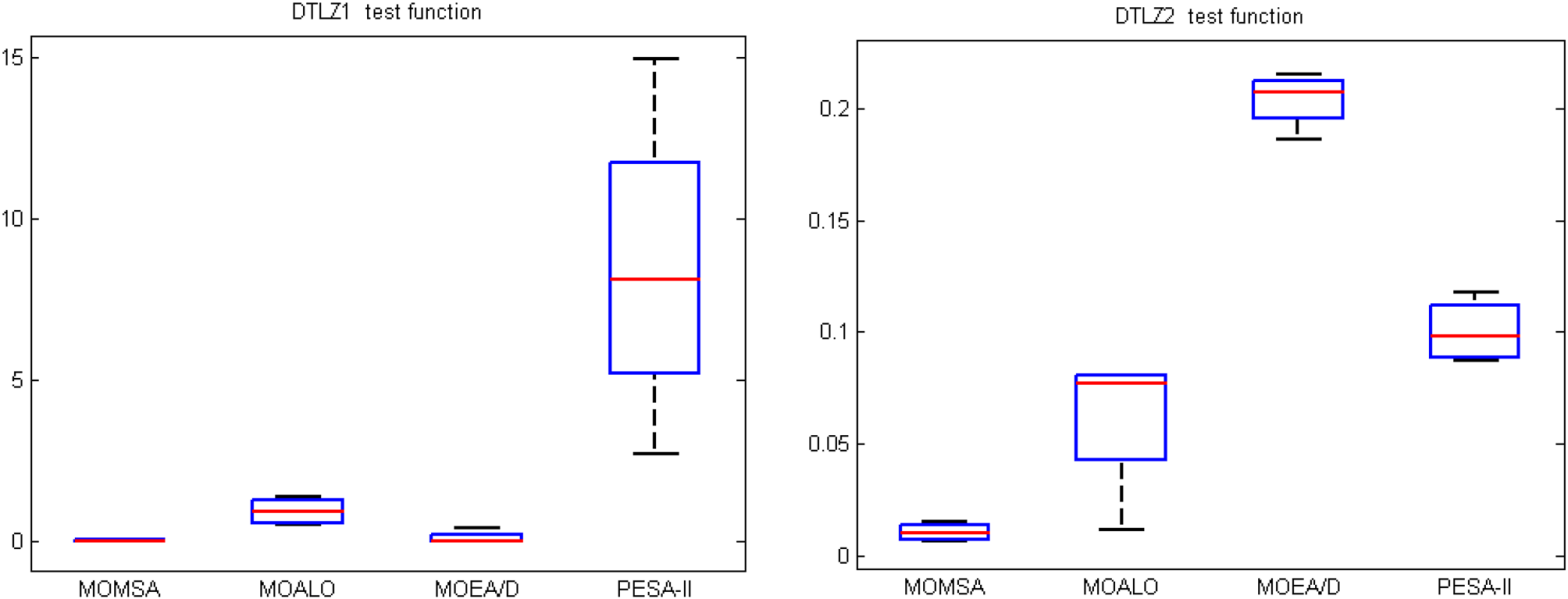
Boxplot of the statistical results for *S* evaluation metrics on DTLZ1 and DTLZ2 tri-objective test function.

For the tri-objective benchmark functions of DTLZ1, as illustrated by Fig. 10, the boxplot of the MOMSA algorithm was super narrow and located under the minima of the other algorithms. After the MOMSA, the MOEA/D demonstrated better results followed by MOALO. The performance of PESA-II was poor. For the DTLZ2 benchmark function, the MOMSA had the lowest and the narrowest boxplot of S metric among all, indicating its highest efficiency in solving the DTLZ2 benchmark function. Similar results were obtained for the performance metrics of *GD*, *MS* and Δ.

## Conclusion

As the superiority of single-objective moth swarm algorithm (MSA) in solving various engineering problems was confirmed by previous studies, this study proposed the multi-objective version of MSA, called MOMSA, to solve various multi-objective problems. Accordingly, a series of improvements was applied in algorithms’ synchronization capability and selection method. The capability of the proposed MOMSA in comparison with three well-known multi-objective algorithms of MOEA/D, PESA-II, and MOALO were tested on 7 standard benchmark functions having 7 to 30 dimensions including five bi-objective ZDT functions and two tri-objective DTLZ functions. The results were evaluated by four performance metrics. It was found that the performance of the proposed MOMSA algorithm became more evident with increasing the dimensions and the complexity of the problem (ZDT1 to ZDT3). So that, in the ZDT1 problem with 30 decision variables, the MOMSA algorithm showed better performance (*GD* = 0.028, *S* = 0.050, Δ = 0.696, *MS* = 0.986) than the MOEA/D algorithm (*GD* = 0.159, *S* = 0.051, Δ = 1.044, *MS* = 1.518), and the MOALO algorithm (*GD* = 0.044, S = 0.071, Δ = 1.345, *MS* = 0.499), and the PESA-II algorithm (*GD* = 0.079, S = 0.068, Δ = 0.763, *MS* = 0.916). Also, the Pareto front derived by the MOMSA algorithm in most cases was more regular and at a more appropriate level than the MOAE/D, MOALO and SPEA-II algorithms. The results indicated the superior performance of the MOMSA and the MOEA/D algorithms over the other two algorithms in both the ZDT and DTLZ benchmark functions. The results also showed that, the dispersion of MOMSA and MOEA/D algorithm distances was smaller than the MOALO and the SPEA-II algorithms with theoretical solutions to the almost all of the ZDT and DTLZ benchmark functions. In addition to the strong convergence to the exact Pareto front, the proposed MOMSA had less dispersion in solutions and achieved the desirable solution in most of the benchmark problems. Regarding the impressive results of the MOMSA algorithm, this study recommends it as a robust and reliable multi-objective optimization model for various optimization problems.
